# Supplementary material for: BRCA2 stabilises RAD51 and DMC1 nucleoprotein filaments through a conserved interaction mode
Source: Nat Commun. 2024 Sep 27;15:8292. doi: 10.1038/s41467-024-52699-3 (PMC11436757; doi:10.1038/s41467-024-52699-3)
Supplement: Supplementary file 1 — Supplementary Information [file 41467_2024_52699_MOESM1_ESM.pdf]

Supplementary Information

BRCA2 stabilises RAD51 and DMC1 nucleoprotein filaments through a conserved interaction mode

James M. Dunce and Owen R. Davies

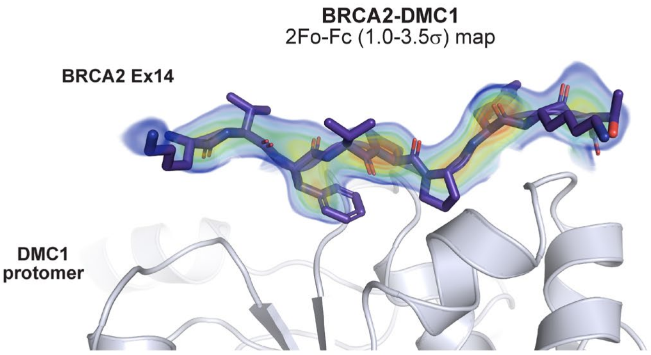

Supplementary Figure 1

Crystal structure of the BRCA2-DMC1 core complex.

2Fo-Fc electron density map of the BRCA2-DMC1 core structure, presented as a rainbow between 1.0σ (blue) and 3.5σ (red), superimposed on the refined crystallographic model.

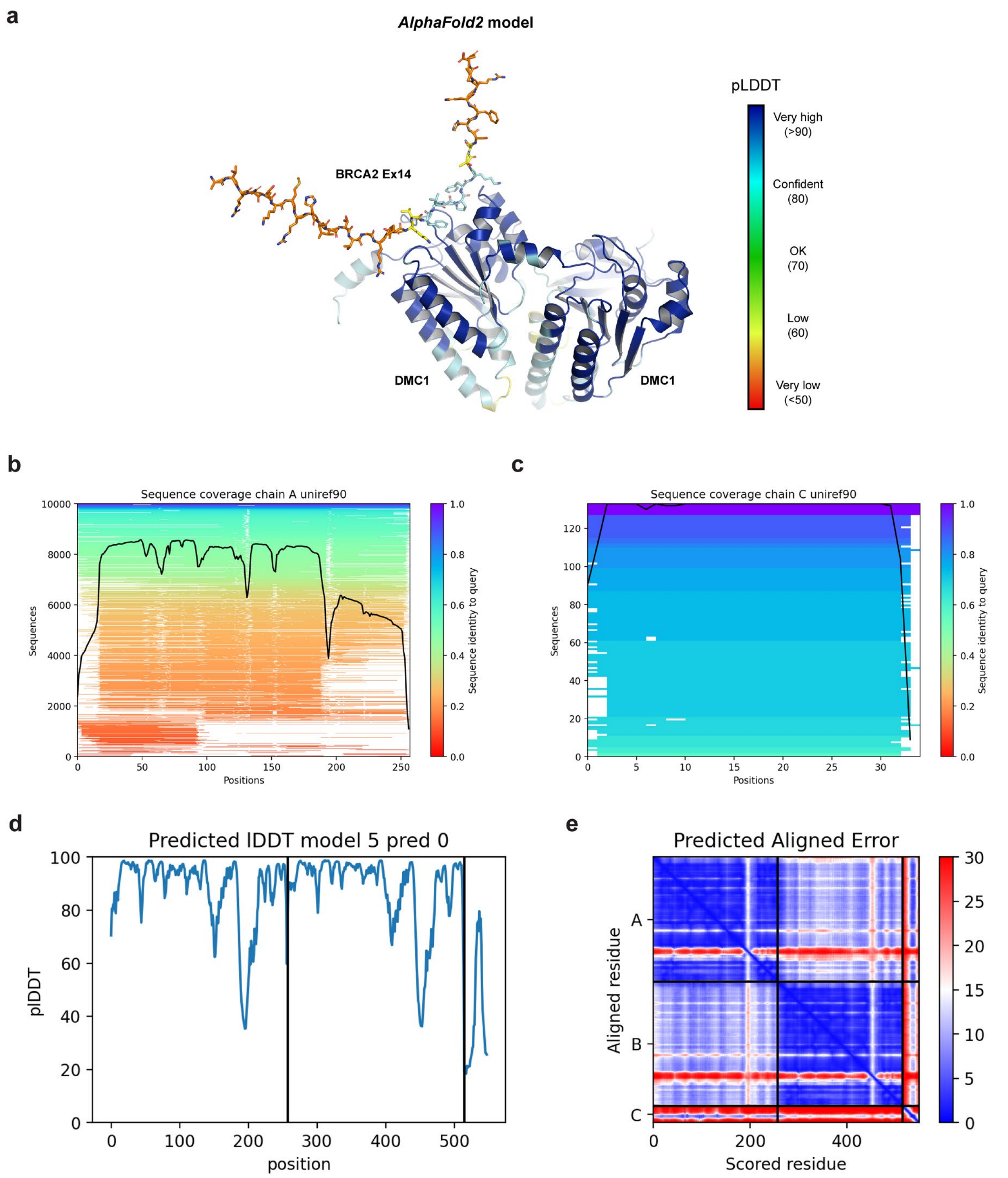

Supplementary Figure 2

***AlphaFold2* model of the BRCA2 Ex14-DMC1 core 1:2 complex.**

(**a**) *AlphaFold2* model of the BRCA2 Ex14-DMC1 ΔN 1:2 complex coloured according to predicted LDDT (pLDDT) scores, between blue (>90) and red (<50). (**b**) Representations of the multiple sequence alignments generated and used by *AlphaFold2*, showing the number of sequences and sequence identity against the position along the (**b**) DMC1 ΔN and (**c**) BRCA2 Ex14 query sequences. (**d**) Predicted

LDDT (pLDDT) scores shown for each amino-acid of the two DMC1 ΔN and one BRCA2 Ex14 chains. (**e**) Predicted aligned error scores between each amino-acid of the DMC1 ΔN and BRCA2 Ex14 chains, between blue (low error) and red (high error).

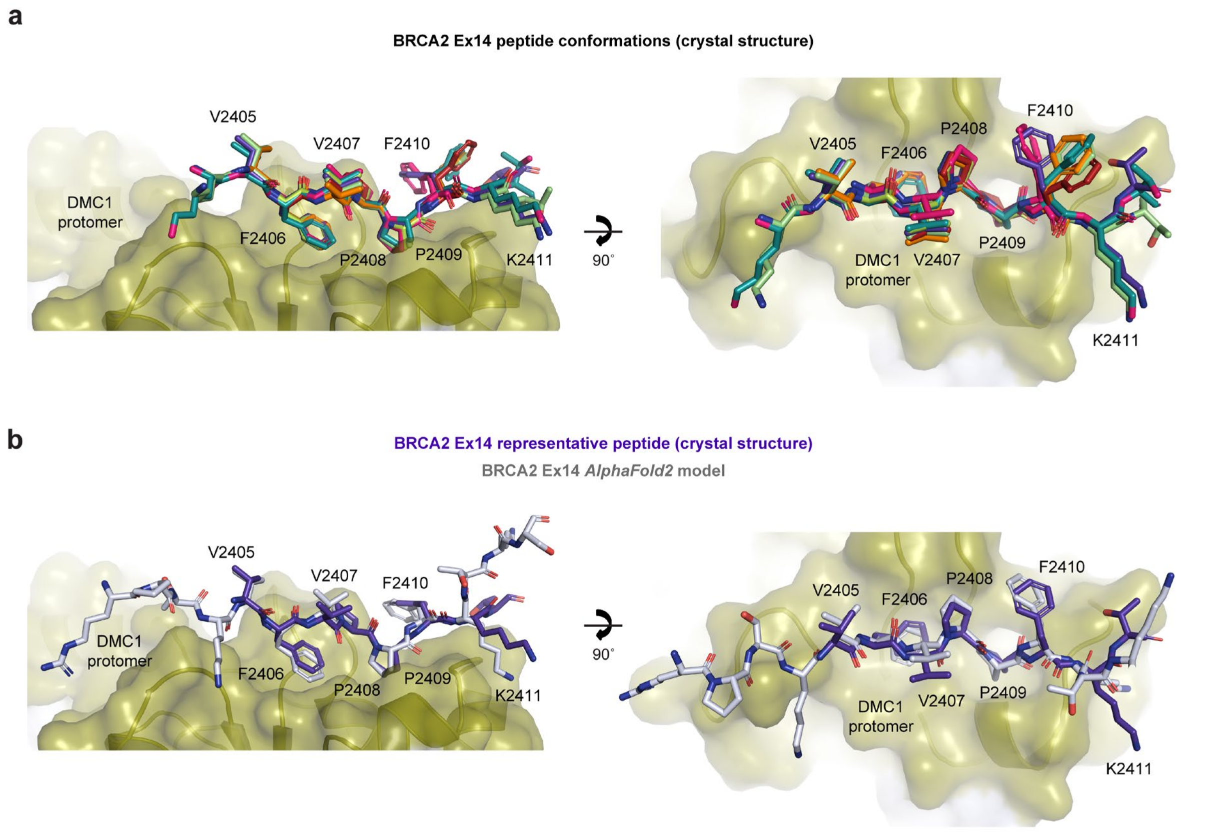

Supplementary Figure 3

Conformation of Ex14 within the BRCA2-DMC1 core crystal structure.

(**a**) Superposition of the seven Ex14 peptides that were built into electron density of the BRCA2-DMC1 crystal structure, aligned via their bound DMC1 protomers. (**b**) Superposition of a representative Ex14 peptide of the BRCA2-DMC1 structure (purple) and the DMC1-Ex14 *AlphaFold2* model (Supplementary Figure 2), aligned via their bound DMC1 protomers.

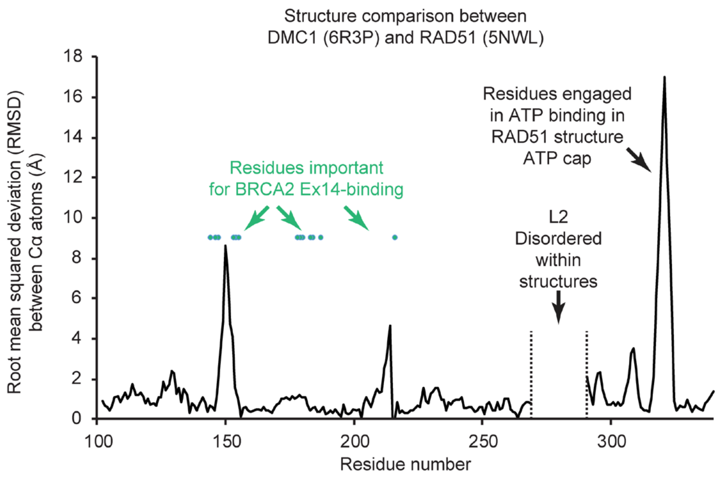

Supplementary Figure 4

Conservation of sequences within DMC1 and RAD51 recombinases.

Root mean squared deviation (RMSD) between Cα atoms of DMC1 and RAD51, using structures 6R3P (this study) and 5NWL[72].

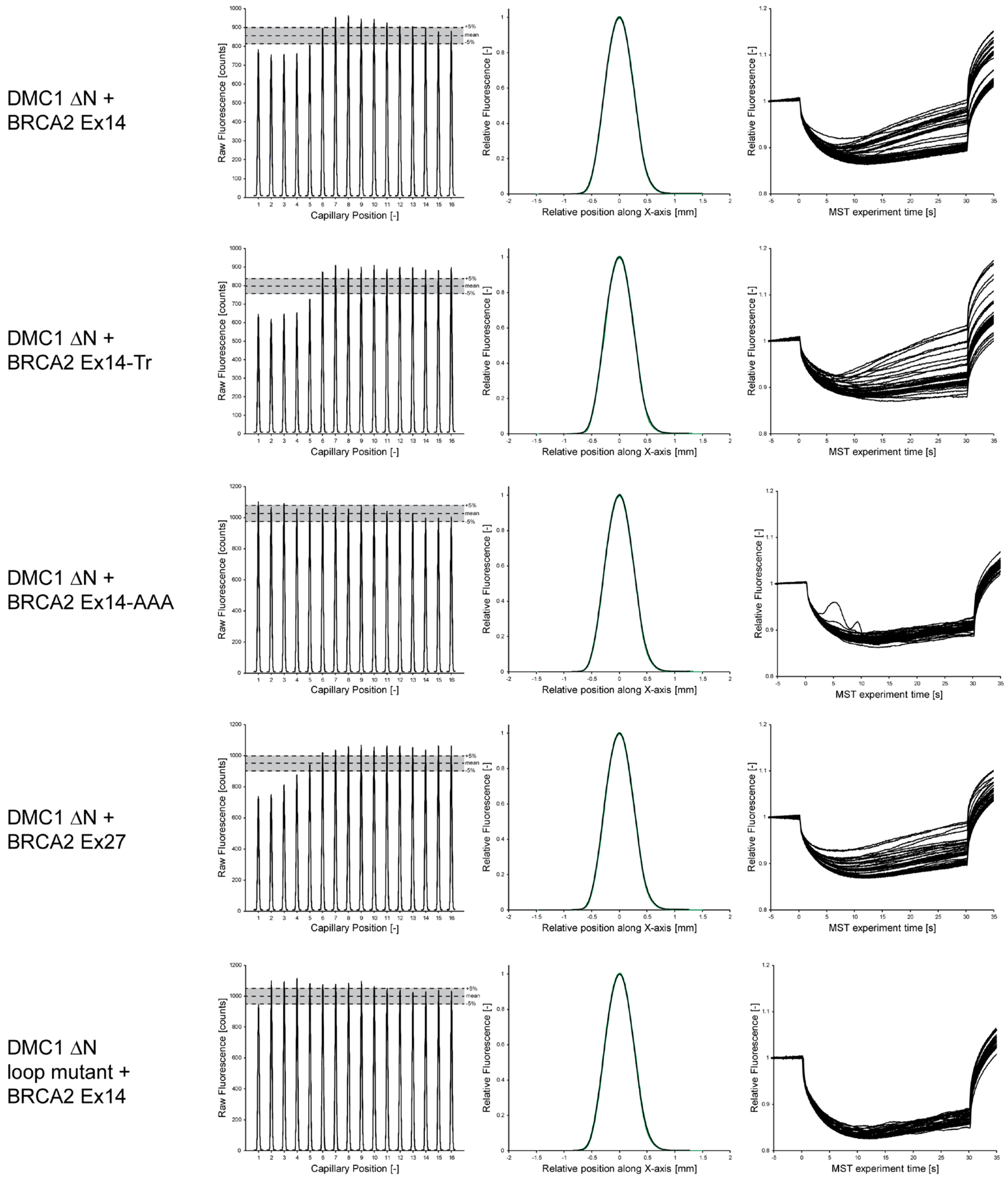

Supplementary Figure 5

Microscale thermophoresis (MST) analysis of the BRCA2 Ex14-DMC1 interaction.

MST analysis corresponding to Figure 2a. Initial fluorescence readings indicating a ligand-induced change in initial fluorescence (left), capillary scans of symmetrical shape indicate that there is no sample adsorbance to the capillaries (middle) and microscale thermophoresis traces for each of the

data series (right), for each BRCA2 peptide and DMC1 ΔN construct, as indicated. Source data are provided as a Source Data file.

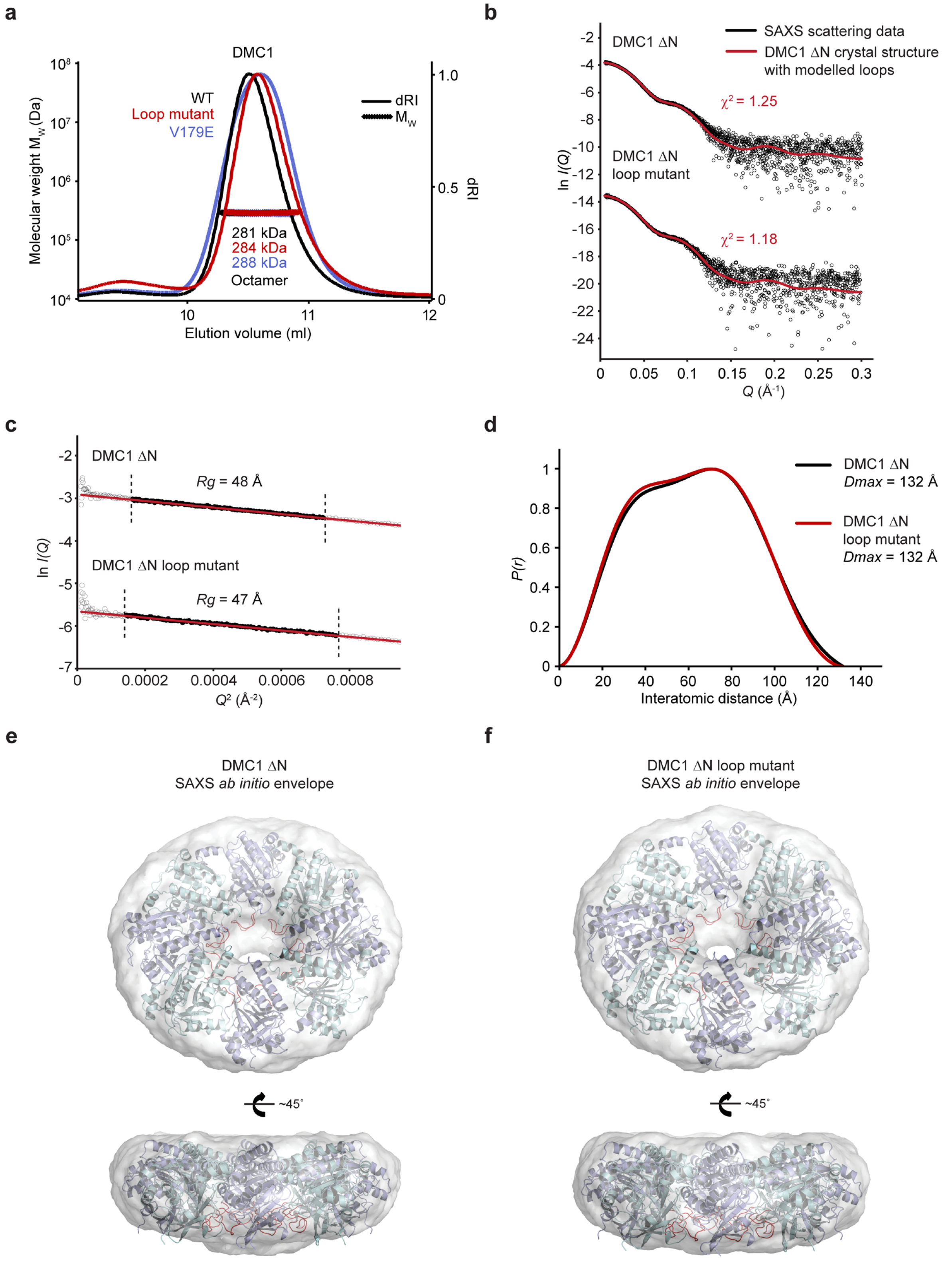

Supplementary Figure 6

Solution structure of DMC1 ΔN WT, V179E and loop mutants.

(**a**) SEC-MALS analysis of DMC1 wild-type (WT; black), loop mutant (red) and V179E mutant (blue), showing differential refractive index (dRI; solid lines) profiles with fitted molecular weights (*Mw*;

diamonds) across elution peaks. DMC1 WT, loop and V179E mutants form octamers of 281 kDa, 284 kDa and 288 kDa, respectively (theoretical octamers – 301 kDa, 301 kDa and 302 kDa). (**b-f**) SEC-SAXS analysis of DMC1 ΔN wild-type and loop mutant. (**b**) SEC-SAXS data in which the DMC1 ΔN and DMC1 ΔN loop mutant scattering curves are overlaid with the theoretical scattering curve of the DMC1 ΔN crystal structure (PDB accession 6R3P) in which missing loops were added using the *ModLoop* module of *MODELLER* (red), with χ^2 values of 1.25 and 1.18, respectively. (**c**) SAXS Guinier analysis of DMC1 ΔN and DMC1 ΔN loop mutant to determine the radius of gyration (*Rg*); linear fits are shown in red, with the fitted data range highlighted in black and demarcated by dashed lines. The *Q.Rg* values were < 1.3 and *Rg* values were calculated as 48 Å and 47 Å. (**d**) SAXS *P(r)* interatomic distance distributions of DMC1 ΔN (black) and DMC1 ΔN loop mutant (red), showing maximum dimensions (*Dmax*) of 132 Å. (**e,f**) SAXS *ab initio* models of (**e**) DMC1 ΔN and (**f**) DMC1 ΔN loop mutant. 15 and 12 independent *GASBOR* models were averaged and shown with the DMC1 ΔN crystal structure (PDB accession 6R3P) docked into the SAXS envelopes.

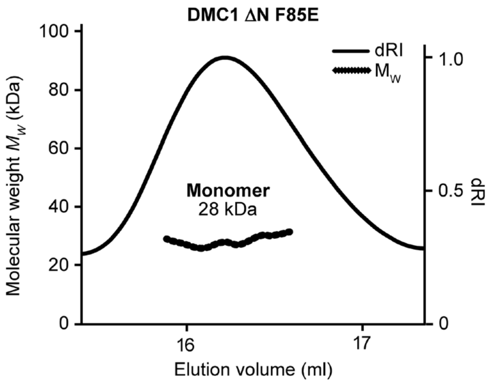

Supplementary Figure 7

SEC-MALS analysis of DMC1 ΔN F85E.

SEC-MALS analysis of DMC1 ΔN F85E, showing differential refractive index (dRI; solid lines) profiles with fitted molecular weights (*Mw*; diamonds) across elution peaks. DMC1 ΔN F85E forms a 28 kDa monomer (theoretical monomer – 29 kDa).

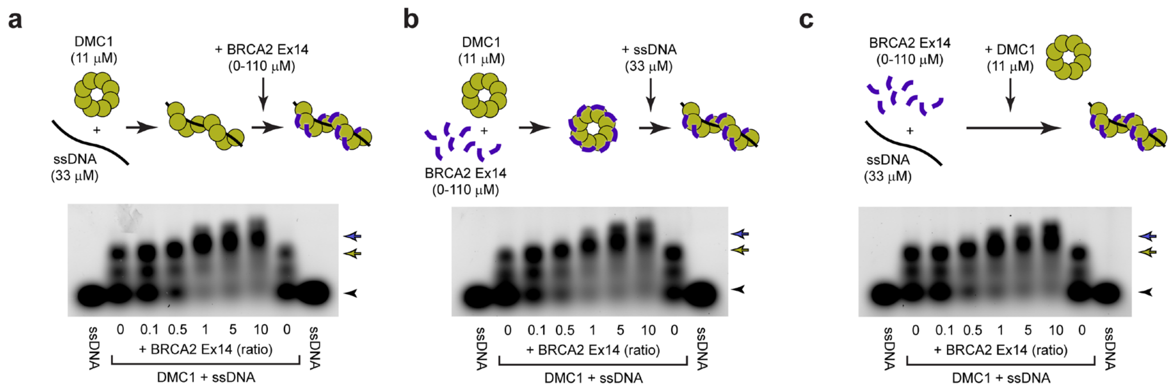

Supplementary Figure 8

Stimulation of DMC1-ssDNA filament formation by Ex14 in different orders of addition.

(**a-c**) EMSAs analysing the ability of BRCA2 Ex14 to bind and promote the formation of DMC1-ssDNA filaments. These were performed using TAE pH 7.5 conditions in which DMC1-ssDNA binding is initially incomplete, and is then promoted with super-shift in the presence of Ex14 (corresponding to Figure 3a). Similar patterns of DMC1-ssDNA filament formation and super-shift were observed when (**a**) Ex14 was added after DMC1-ssDNA incubation, (**b**) Ex14 was incubated with DMC1 prior to the addition of ssDNA, and (**c**) Ex14 was incubated with ssDNA prior to the addition of DMC1. BRCA2 peptide concentrations are shown as molar ratios with respect to DMC1 protomers. Arrowheads, free ssDNA; yellow arrows, DMC1-ssDNA complexes; blue arrows, BRCA2-DMC1-ssDNA complexes. The ssDNA substrate is a 100-nucleotide random sequence (provided in Methods). Source data are provided as a Source Data file.

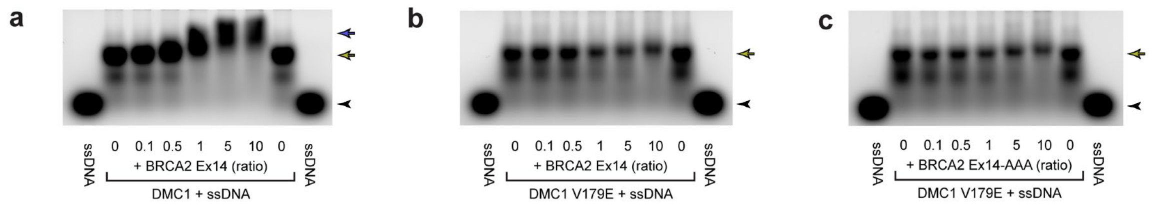

Supplementary Figure 9

Super-shift of DMC1-ssDNA filaments by Ex14 using DMC1 WT and V179E mutant

(**a-c**) EMSAs analysing the ability of BRCA2 Ex14 to bind and promote the formation of nucleoprotein filaments formed between WT or V179E DMC1 and ssDNA. These were performed using TEA pH 7.5 + KCl conditions in which DMC1-ssDNA binding is largely complete and then undergoes super-shift in the presence of Ex14 (corresponding to Figure 3b). (**a**) Wild-type DMC1-ssDNA filaments underwent clear super-shift upon addition of Ex14. (**b,c**) DMC1 V179E formed filaments with ssDNA that demonstrated only minimal super-shift upon addition of (**b**) Ex14 or (**c**) Ex14-AAA peptides. BRCA2 peptide concentrations are shown as molar ratios with respect to DMC1 protomers. Arrowheads, free ssDNA; yellow arrows, DMC1-ssDNA complexes; blue arrows, BRCA2-DMC1-ssDNA complexes. The ssDNA substrate is a 100-nucleotide random sequence (provided in Methods). Source data are provided as a Source Data file.

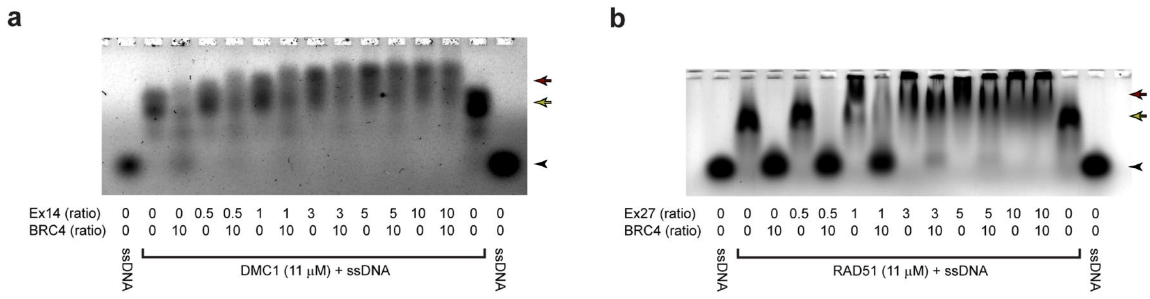

Supplementary Figure 10

Protection of recombinase-ssDNA filaments by Ex14 and Ex27 at different molar ratios.

(**a,b**) EMSAs analysing the ability of BRCA2 Ex14 and Ex27 to protect DMC1-ssDNA and RAD51-ssDNA filaments from BRC4-induced disruption. BRCA2 peptide concentrations are shown as molar ratios with respect to DMC1 protomers. Arrowheads, free ssDNA; yellow arrows, DMC1/RAD51-ssDNA complexes; red arrows, BRCA2-DMC1/RAD51-ssDNA complexes. (**a**) EMSAs using TEA pH 7.5 + KCl conditions in which DMC1-ssDNA binding is disrupted by a 10-fold stoichiometric excess of BRC4. Reactions were performed upon pre-incubation with Ex14, indicating that some protection is conferred by an equimolar concentration of Ex14, and is complete at between a 5- and 10-fold molar excess of Ex14. The ssDNA substrate is a 100-nucleotide random sequence (provided in Methods). (**b**) EMSAs in which RAD51 DMC1-ssDNA binding is disrupted by a 10-fold stoichiometric excess of BRC4. Reactions were performed upon pre-incubation with Ex27, indicating that some protection is conferred by a 3-fold molar excess of Ex27, and is complete at a 10-fold molar excess of Ex27. The ssDNA substrate is a 100-nucleotide poly-dT sequence. Source data are provided as a Source Data file.

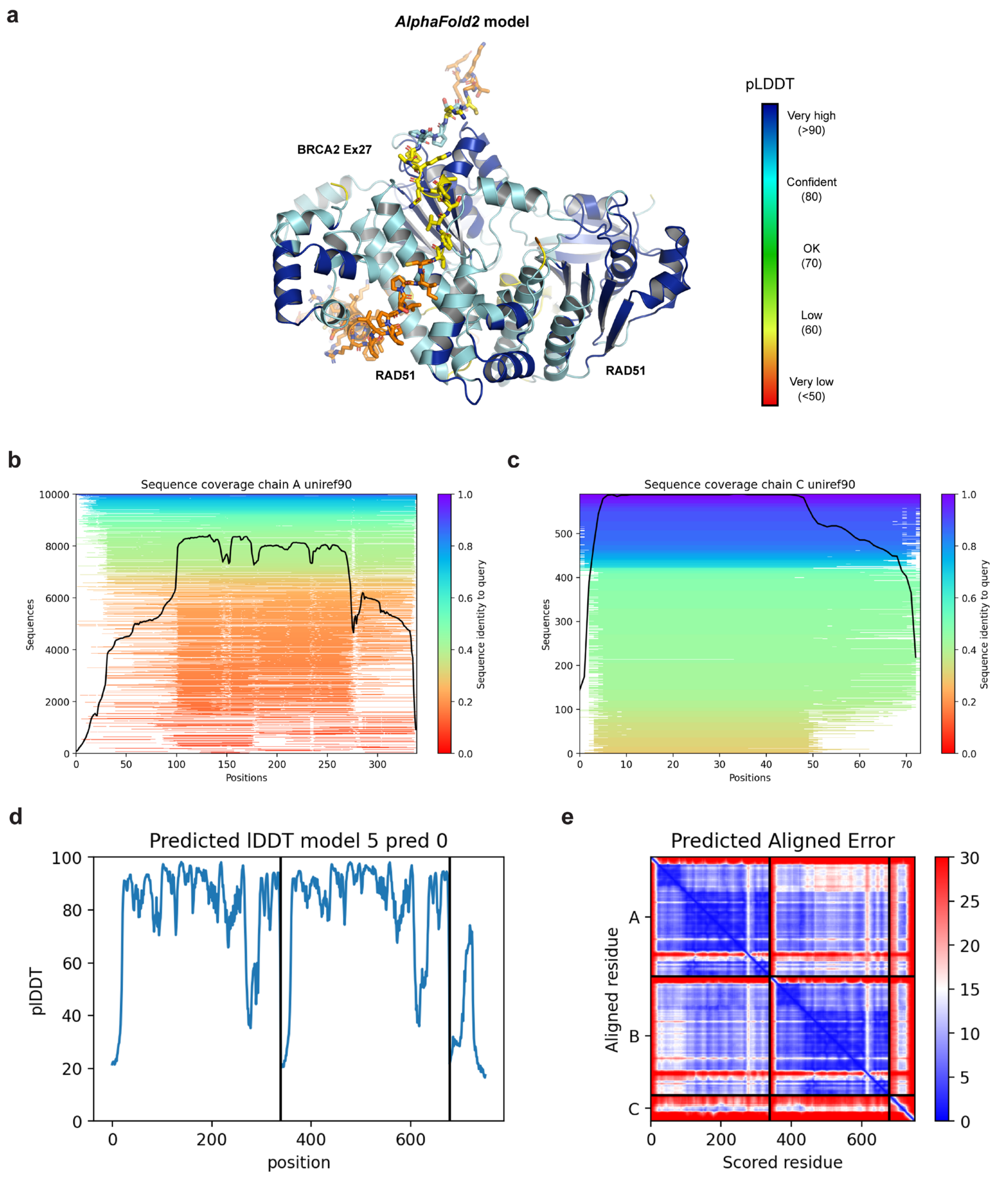

Supplementary Figure 11

***AlphaFold2* model of the BRCA2 Ex27-RAD51 ΔN 1:2 complex.**

(**a**) *AlphaFold2* model of the BRCA2 Ex27-RAD51 ΔN 1:2 complex coloured according to predicted LDDT (pLDDT) scores, between blue (>90) and red (<50). (**b**) Representations of the multiple sequence alignments generated and used by *AlphaFold2*, showing the number of sequences and sequence

identity against the position along the (**b**) RAD51 and (**c**) BRCA2 Ex27 query sequences. (**d**) Predicted LDDT (pLDDT) scores shown for each amino-acid of the two RAD51 and one BRCA2 Ex27 chains. (**e**) Predicted aligned error scores between each amino-acid of the two RAD51 and one BRCA2 Ex27 chains, between blue (low error) and red (high error).

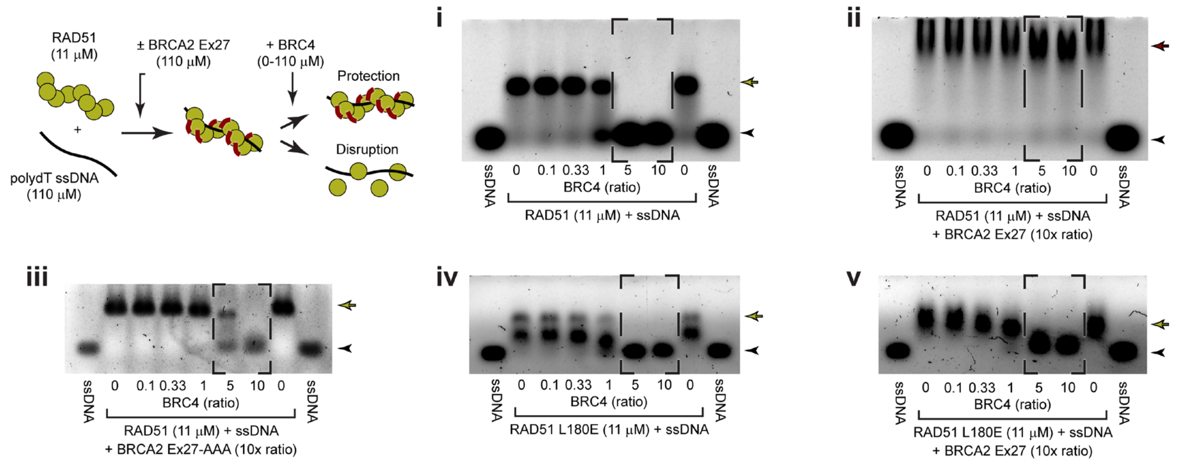

Supplementary Figure 12

Protection of the RAD51-ssDNA (polydT) filaments by Ex27.

EMSAs analysing the ability of BRCA2 Ex27 to protect RAD51-ssDNA filaments from BRC4-induced disruption using a 100-nucleotide polydT ssDNA substrate. (**i**) RAD51-ssDNA (polydT) binding is disrupted by a stoichiometric excess of BRC4. (**ii**) BRCA2 Ex27 but not (**iii**) BRCA2 Ex14-AAA mutant protects against BRC4-mediated disruption (dashed, boxed), and (**iv**) RAD51 loop mutant L180E undergoes similar disruption, but (**v**) is not protected by BRCA2 Ex27. BRCA2 peptide concentrations are shown as molar ratios with respect to RAD51 protomers. Arrowheads, free ssDNA; yellow arrows, RAD51-ssDNA complexes; red arrows, BRCA2-RAD51-ssDNA complexes. These correspond to the EMSAs performed using 100-nucleotide random sequence ssDNA, shown in Figure 4e, in which BRC4-induced disruption occurred at equimolar ratios and protection was less complete. Hence, RAD51-ssDNA filaments are more stable and protection by Ex27 is more complete for polydT than random sequence substrates. Source data are provided as a Source Data file.

Supplementary Table 1

SEC-SAXS data table.

(*a*) Sample details

	DMC1 ΔN	DMC1 ΔN loop mutant
Organism	*H. sapiens*	*H. sapiens*
Description: sequence (including Uniprot ID + uncleaved tags), bound ligands/modifications, *etc.*	GSMPGFLTAFEYSEKRKMVFHITTGSQ EFDKLLGGGIESMAITEAFGEFRTGKT QLSHTLCVTAQLPGAGGYPGGKIIFIDT ENTFRPDRLRDIADRFNVDHDAVLDN VLYARAYTSEHQMELLDYVAAKFHEE AGIFKLLIIDSIMALFRVDFSGRGELAER QQKLAQMLSRLQKISEEYNVAVFVTN QMTADPGATMTFQADPKKPIGGHIL AHASTTRISLRKGRGELRIAKIYDSPEM PENEATFAITAGGIGDAK	GSMPGFLTAFEYSEKRKMVFHITTGSQE FDKLLGGGIESMAITEAFGEFRTGKTQL SHTLCVTAQLPIDRGGGEGKIIFIDTENT FRPDRLRDIADRFGLSGSDVLDNVLYAR AYTSEHQMELLDYVAAKFHEEAGIFKLLI IDSIMALFRVDFSGRGELAERQQKLAQ MLSRLQKISEEYNVAVFVTNQMTADPG ATMTFQADPKKPIGGHILAHASTTRISL RKGRGELRIAKIYDSPEMPENEATFAITA GGIGDAK
Extinction coefficient ε (280 nm; M^{-1} cm^{-1})	10430	8940
Molecular mass *M* from chemical composition (Da)	28703.70	28693.70
For SEC-SAS, loading volume/concentration, (mg ml^{-1})	6 mg ml^{-1}<br>100 μl	5 mg ml^{-1}<br>100 μl
injection volume (μl), flow rate (ml min^{-1})	0.5 ml min^{-1}	0.5 ml min^{-1}
Solvent composition and source	20 mM Tris pH 8.0, 150 mM KCl	20 mM Tris pH 8.0, 150 mM KCl

(*b*) SAS data collection parameters

Source, instrument and description or reference: Beamline B21 of Diamond Light Source

Wavelength (Å): 0.99987 Å

Beam geometry (size, sample-to-detector distance): 1.1 x 0.2 mm cross-section, detector distance 4.014 m

q-measurement range ($Å^{-1}$ or nm^{-1}): $Å^{-1}$

Exposure time, number of exposures: 3.0 s exposure time; 1200 exposures

Sample temperature (ºC): 22ºC

(*c*) Software employed for SAS data reduction, analysis and interpretation

SAS data reduction to sample–solvent scattering: *ScÅtter* 3.0

Basic analyses: Guinier, *P*(*r*), scattering particle volume (*e.g.* Porod volume V_P or volume of correlation V_c): *PRIMUS*

Shape/bead modelling: *GASBOR*

Molecular graphics: *PyMOL*

(*d*) Structural parameters

Guinier Analysis	DMC1 ΔN	DMC1 ΔN loop mutant
I(0) (cm^{-1})	0.116	0.052
R_g (Å)	48	47
q-range ($Å^{-1}$)	0.004 – 0.3	0.004 – 0.3
P(*r*) analysis	DMC1 ΔN	DMC1 ΔN loop mutant
I(0) (cm^{-1})	0.120	0.051
R_g (Å)	48	47
d_{max} (Å)	132	132
q-range ($Å^{-1}$)	0.004 – 0.3	0.004 – 0.3
Quality-of-fit parameter	0.9013	0.9122
Volume (V_P)	359696	354754

(e) Shape modelling results (a complete panel for each method)

	DMC1 ΔN	DMC1 ΔN loop mutant
q-range for fitting	0.005 – 0.1695	0.005 – 0.1654
Symmetry/anisotropy assumptions	P8	P8
χ^2 value/range	0.9798 - 1.129	1.109 - 1.165
