# Supplementary material for: BRCA2 stabilises RAD51 and DMC1 nucleoprotein filaments through a conserved interaction mode
Source: Nat Commun. 2024 Sep 27;15:8292. doi: 10.1038/s41467-024-52699-3 (PMC11436757; doi:10.1038/s41467-024-52699-3)
Supplement: Supplementary file 3 — Reporting Summary [file 41467_2024_52699_MOESM3_ESM.pdf]

nature portfolio

Corresponding author(s): Prof. Owen Richard Davies

Last updated by author(s): Sep 4, 2024

Reporting Summary

Nature Portfolio wishes to improve the reproducibility of the work that we publish. This form provides structure for consistency and transparency in reporting. For further information on Nature Portfolio policies, see our Editorial Policies and the Editorial Policy Checklist.

Statistics

For all statistical analyses, confirm that the following items are present in the figure legend, table legend, main text, or Methods section.

n/a	Confirmed	
☐	☒	The exact sample size (*n*) for each experimental group/condition, given as a discrete number and unit of measurement
☒	☐	A statement on whether measurements were taken from distinct samples or whether the same sample was measured repeatedly
☒	☐	The statistical test(s) used AND whether they are one- or two-sided<br>*Only common tests should be described solely by name; describe more complex techniques in the Methods section.*
☒	☐	A description of all covariates tested
☒	☐	A description of any assumptions or corrections, such as tests of normality and adjustment for multiple comparisons
☒	☐	A full description of the statistical parameters including central tendency (e.g. means) or other basic estimates (e.g. regression coefficient) AND variation (e.g. standard deviation) or associated estimates of uncertainty (e.g. confidence intervals)
☒	☐	For null hypothesis testing, the test statistic (e.g. *F*, *t*, *r*) with confidence intervals, effect sizes, degrees of freedom and *P* value noted<br>*Give P values as exact values whenever suitable.*
☒	☐	For Bayesian analysis, information on the choice of priors and Markov chain Monte Carlo settings
☒	☐	For hierarchical and complex designs, identification of the appropriate level for tests and full reporting of outcomes
☒	☐	Estimates of effect sizes (e.g. Cohen's *d*, Pearson's *r*), indicating how they were calculated

Our web collection on statistics for biologists contains articles on many of the points above.

Software and code

Policy information about availability of computer code

Data collection	ASTRA® 6 software<br>GE Healthcare Unicorn 6<br>MO.Control (NanoTemper Technologies) - MST Data Collection

Data analysis	XDS - X-ray diffraction data processing<br>AutoPROC - X-ray diffraction data processing<br>CCP4 Aimless - X-ray diffraction data merging<br>Phaser - Crystallographic molecular replacement<br>PHENIX - Crystallographic experimental phasing, model building and refinement<br>Coot - Crystallographic model visualisation and manual building<br>PyMOL version 2.4 - Molecular graphics software<br>Adobe Photoshop 2022 - image processing<br>Adobe Illustrator 2022 - vector graphics for figures<br>Alphafold2 - molecular modelling<br>MODELLE - molecualr modelling<br>ASTRA® 6 software - MALS data analysis<br>ScÅtter 3.0 - SAXS data analysis<br>ATSAS software suite (PRIMUS, CRYSOL, GASBOR) - SAXS data analysis<br>MO.AffinityAnalysis (NanoTemper Technologies) - MST Data Analysis

For manuscripts utilizing custom algorithms or software that are central to the research but not yet described in published literature, software must be made available to editors and reviewers. We strongly encourage code deposition in a community repository (e.g. GitHub). See the Nature Portfolio guidelines for submitting code & software for further information.

Data

Policy information about availability of data

All manuscripts must include a data availability statement. This statement should provide the following information, where applicable:

- Accession codes, unique identifiers, or web links for publicly available datasets
- A description of any restrictions on data availability
- For clinical datasets or third party data, please ensure that the statement adheres to our policy

Crystallographic structure factors and atomic co-ordinates have been deposited in the Protein Data Bank (PDB) under accession numbers 6R3P [https://doi.org/10.2210/pdb6R3P/pdb] and 8R2G [https://doi.org/10.2210/pdb8R2G/pdb], and raw diffraction data have been uploaded to https://proteindiffraction.org/. This study used PDB entries 1N0W [https://doi.org/10.2210/pdb1N0W/pdb], 4HYY [https://doi.org/10.2210/pdb4HYY/pdb], 7C9C [https://doi.org/10.2210/pdb7C9C/pdb] and 8BSC [https://doi.org/10.2210/pdb8BSC/pdb]. Data that support the findings of this study are provided in the accompanying supplementary information and source data files. Source data are provided with this paper.

Research involving human participants, their data, or biological material

Policy information about studies with human participants or human data. See also policy information about sex, gender (identity/presentation), and sexual orientation and race, ethnicity and racism.

Reporting on sex and gender	N/A
Reporting on race, ethnicity, or other socially relevant groupings	N/A
Population characteristics	N/A
Recruitment	N/A
Ethics oversight	N/A

Note that full information on the approval of the study protocol must also be provided in the manuscript.

Field-specific reporting

Please select the one below that is the best fit for your research. If you are not sure, read the appropriate sections before making your selection.

☒ Life sciences ☐ Behavioural & social sciences ☐ Ecological, evolutionary & environmental sciences

For a reference copy of the document with all sections, see nature.com/documents/nr-reporting-summary-flat.pdf

Life sciences study design

All studies must disclose on these points even when the disclosure is negative.

Sample size	The volume and concentration of protein samples analysed in biochemical and biophysical analyses were selected to provide sufficient signal-to-noise for accurate data analysis. Protein concentration in crystallisation experiments was as required for crystal growth.
Data exclusions	No data were excluded from analyses.

Replication	All biochemical and biophysical experiments were repeated at least three times with separately prepared recombinant protein material. All attempts at replication were successful.
Randomization	Protein samples were not grouped into experimental groups as this is not required for the study.
Blinding	Investigators were not blinded as this is not applicable to our study.

Reporting for specific materials, systems and methods

We require information from authors about some types of materials, experimental systems and methods used in many studies. Here, indicate whether each material, system or method listed is relevant to your study. If you are not sure if a list item applies to your research, read the appropriate section before selecting a response.

Materials & experimental systems

n/a	Involved in the study
☒	☐ Antibodies
☒	☐ Eukaryotic cell lines
☒	☐ Palaeontology and archaeology
☒	☐ Animals and other organisms
☒	☐ Clinical data
☒	☐ Dual use research of concern
☒	☐ Plants

Methods

n/a	Involved in the study
☒	☐ ChIP-seq
☒	☐ Flow cytometry
☒	☐ MRI-based neuroimaging
